# Engineered Plant‐Based Nanocellulose Hydrogel for Small Intestinal Organoid Growth

**DOI:** 10.1002/advs.202002135

**Published:** 2020-11-20

**Authors:** Rodrigo Curvello, Genevieve Kerr, Diana J. Micati, Wing Hei Chan, Vikram S. Raghuwanshi, Joseph Rosenbluh, Helen E. Abud, Gil Garnier

**Affiliations:** ^1^ Bioresource Processing Research Institute of Australia (BioPRIA) Department of Chemical Engineering Monash University Clayton Victoria 3800 Australia; ^2^ Department of Anatomy and Developmental Biology and Development and Stem Cells Program Monash Biomedicine Discovery Institute Clayton Victoria 3800 Australia; ^3^ Department of Biochemistry and Molecular Biology Monash University Clayton Victoria 3800 Australia

**Keywords:** hydrogels, nanocellulose, organoids, rheology, transcriptomic profile

## Abstract

Organoids are three‐dimensional self‐renewing and organizing clusters of cells that recapitulate the behavior and functionality of developed organs. Referred to as “organs in a dish,” organoids are invaluable biological models for disease modeling or drug screening. Currently, organoid culture commonly relies on an expensive and undefined tumor‐derived reconstituted basal membrane which hinders its application in high‐throughput screening, regenerative medicine, and diagnostics. Here, we introduce a novel engineered plant‐based nanocellulose hydrogel is introduced as a well‐defined and low‐cost matrix that supports organoid growth. Gels containing 0.1% nanocellulose fibers (99.9% water) are ionically crosslinked and present mechanical properties similar to the standard animal‐based matrix. The regulation of the osmotic pressure is performed by a salt‐free strategy, offering conditions for cell survival and proliferation. Cellulose nanofibers are functionalized with fibronectin‐derived adhesive sites to provide the required microenvironment for small intestinal organoid growth and budding. Comparative transcriptomic profiling reveals a good correlation with transcriptome‐wide gene expression pattern between organoids cultured in both materials, while differences are observed in stem cells‐specific marker genes. These hydrogels are tunable and can be combined with laminin‐1 and supplemented with insulin‐like growth factor (IGF‐1) to optimize the culture conditions. Nanocellulose hydrogel emerges as a promising matrix for the growth of organoids.

## Graphical abstract

Plant‐based nanocellulose hydrogel is introduced as a well‐defined and very low‐cost porous nanofibrous matrix that supports organoid growth. The mechanical, chemical, and biological properties of the gel are engineered to mimic the extracellular matrix (ECM), providing the required microenvironment for small intestinal organoid culture. This performant hydrogel is tunable with ECM‐derived components, emerging as a promising biomaterial for organoid systems.

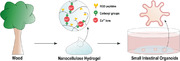

## Introduction

Organoids are complex 3D cell culture systems able to self‐renew and reorganize in vitro. These “mini‐organs” mimic many of the physiological properties of tissues and organs including much of their behavior and functionality.^[^ ^1^, ^2^ ^]^ These attributes make organoids a robust and reliable biological model for many applications in biomedicine, especially studies on drug screening and design, disease modeling, and developmental biology.^[^ ^3^ ^]^ Organoids can be generated from embryonic, adult, pluripotent or induced pluripotent stem cells (ESC, ASC, PSC, or iPSC, respectively), as well as from primary healthy or cancerous tissues.^[^ ^1^, ^4^ ^]^ For establishment and long‐term culture, organoids are commonly embedded within an Engelbreth–Holm Swarm (EHS) matrix derived from the reconstituted basement membrane of mouse sarcoma.^[^ ^5^, ^6^ ^]^ This decellularized extracellular matrix (ECM)‐based gel is known as Matrigel (Corning), Cultrex BME (Trevigen), or Geltrex (Gibco). In spite of its wide application in organoids culture, the EHS matrix has a poorly defined composition and an important batch to batch variability. Indeed, more than a thousand unique biomolecules are found in Matrigel and the batch to batch consistency of its proteic components is limited to 50%.^[^ ^7^ ^]^ The lack of understanding of how these factors influence the experimental conditions, such as cells and its microenvironment, has delayed its translation to clinics.^[^ ^8^ ^]^ Modifying the regular matrix stiffness as well as the laborious process to recover cells encapsulated within the gel stand as critical limitations in research and development. Lastly, to facilitate large‐scale experimental studies and use of cultures in clinical environments, a cost‐effective product is required. Therefore, alternative matrices able to reduce the dependency of Matrigel, or fully replace this animal‐based material—without its critical drawbacks—are urgently needed.

**Figure 1:**
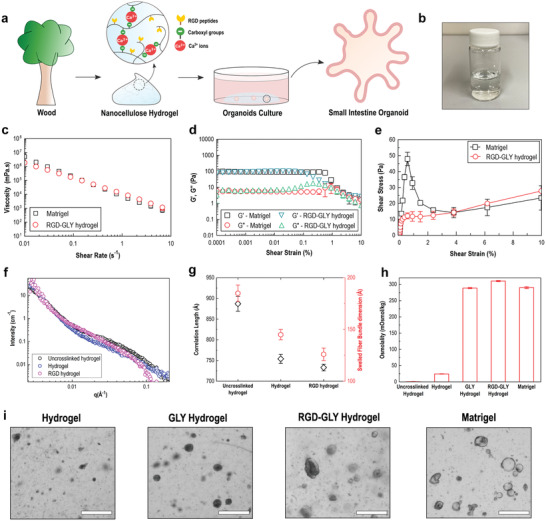
Nanocellulose hydrogel overview and characterization. A) Schematic overview of small intestinal organoid culture in plant‐based nanocellulose hydrogel. B) Nanocellulose hydrogel. C) RGD‐GLY hydrogel presents shear‐thinning behavior similar to Matrigel. D) Storage (G’) and loss modulus (G”) of RGD‐GLY hydrogel also match the viscoelastic rheology of Matrigel. E) Matrigel yields under 55 Pa stress–strain in contrast to RGD‐GLY hydrogel which flows under 10 Pa. F) Effects of Ca^2+^‐mediated crosslinking and RGD grafting on the hydrogel structure are measured by small‐angle neutron scattering (SANS). G) Hydrogel porosity and swelling capacity are affected by both CNF crosslinking and functionalization. H) The osmolality of nanocellulose hydrogel is balanced to 298 mOsmol kg^−1^ by addition of glycine (GLY). I) Crypts seeded in neat nanocellulose hydrogel do not grow. Upon the addition of GLY, the osmolality of the matrix reaches the physiological level and cystic organoids are formed. Nanocellulose functionalization with RGD peptides sustains organoid growth and morphology over 48 h. Scale bars: 100 µm. Results shown represent independent experiments performed in triplicates (*n* = 3, error bars = SD).

In addition to decellularized tissues, natural and synthetic polymeric materials have been explored for organoid systems.^[^ ^9^ ^]^ Cruz‐Acuna et al. reported a four‐armed maleimide‐terminated poly(ethylene glycol) (PEG) hydrogel able to grow human intestinal organoid from ESC and iPSCs.^[^ ^10^ ^]^ Gjoresvski et al. evaluated another PEG‐based matrix with dynamic stiffness where intestinal stem cells expand and form organoids.^[^ ^11^, ^12^ ^]^ These two studies have demonstrated that mechanical properties play a key role in the generation and expansion of organoids in culture. The presence of adhesive sites, mainly Arg‐Gly‐Asp (RGD) peptides, also stands as a fundamental element in these matrices, inducing high cell attachment and differentiation. Similarly, laminin‐1 is suggested to be a required additive to induce robust organoids formation in a fibrin‐based hydrogel.^[^ ^13^ ^]^ However, carbohydrate polymers and plant‐based hydrogels remain poorly explored. Capeling et al. presented a non‐adhesive alginate hydrogel for intestinal organoids growth which was unable to progress into enteroids. In contrast, Wilkinson et al. showed that alginate beads functionalized with type I collagen can support the generation of lung organoids.^[^ ^14^ ^]^ To the best of our knowledge, functionalized cellulose‐based hydrogels have not been rigorously investigated nor successful in growing organoids. Cancerous spheroids (non‐structured clusters of proliferating transformed cells) were previously cultured in neat microfibrilated cellulose scaffolds.^[^ ^15^ ^]^ Similarly, inert cellulose nanofibrillar hydrogel was shown to promote the differentiation of human liver organoids.^[^ ^16^ ^]^ Here, we introduce an engineered plant‐based nanocellulose hydrogel for the growth of mouse small intestinal organoid (**Figure** **1**a).

## Results and Discussion

In a step‐wise approach, the design of the hydrogel is guided to match the biochemical and mechanical properties of the EHS matrix. Oxidized cellulose nanofibers (CNF) are functionalized with fibronectin‐derived moieties (RGD peptides) to enhance cellular interaction between the organoids and the cellulosic surface, inducing adhesion. The temperature‐responsive gellification of Matrigel is mimicked by the ionic crosslinking of CNF. Crypts are suspended in a physically crosslinked hydrogel that undergoes *in situ* Ca^2+^‐mediated ionic crosslinking. The internal remodeling of the hydrogel 3D structure is enabled by the rearrangement of charges and fibers entanglement which are independent of the metalloproteinases expression and activity. This strategy allows the hydrogels not only to reach identical mechanical properties as Matrigel, but also to achieve any level of viscoelastic modulus, whether higher or lower (Figure S1, Supporting Information). The rheological properties of Matrigel (Corning, protein concentration = 8.6 mg mL^−1^) were characterized by the viscosity‐shear, showing a shear‐thinning behavior (Figure 1c). Oscillatory strain sweep of Matrigel measures a storage modulus (G’) of 101 Pa and a loss modulus (G”) of 6 Pa in the linear viscoelastic (LVE) region (Figure 1d). The curves do not exhibit a crossover point, highlighting the dominance of the elastic regime. The Matrigel yielding point occurs at a shear stress of 56 Pa (Figure 1e). Similar to the EHS matrix, nanocellulose hydrogel—here referred to as RGD‐GLY hydrogel (0.1 wt% solids content)—exhibits a characteristic shear‐thinning curve. Measuring the dynamic strain shows RGD‐GLY hydrogel to have an elastic modulus (G’) of 98 Pa, a viscous modulus (G”) of 8 Pa and a yield point of 15 Pa under shear stress. The absence of grafted RGD peptides on the cellulose surface has minimal effects on the material's rheological profile (Figure S2, Supporting Information), whilst hydrogels at higher solids content (0.2–0.5 wt%) form considerably stiffer matrices (Figure S3, Supporting Information) which do not degrade over time (Figure S4, Supporting Information). The remarkable difference between the yielding point of both materials does not show any impact on the culture of organoids, allowing easy recovery of cells by mechanical disruption of the hydrogel, followed by the usual centrifugation when passaged. The material's structure was analyzed under small‐angle neutron scattering (SANS), demonstrating that ionic crosslinking of cellulose nanofibers as well as CNF functionalization modify the hydrogel network organization (Figure 1f). The RGD‐grafted hydrogel porosity is characterized by a correlation length of 730 Å, lower than the 750 Å measured for the non‐functionalized gels (Figure 1g). The structural characterization of the porous hydrogel shows the nanocellulose dimensions to match those of the constitutive collagen fibers present in the EHS matrix.^[^ ^17^ ^]^ These results summed to the rheological measurements show the engineered cellulose gel to have the required mechanical properties.

By opposition to scaffolds for tissue engineering, hydrogels for organoid systems must reconstitute the microenvironment of the basal membrane beyond its stiffness or elasticity. Indeed, cell proliferation as well as cell differentiation are directly dependent on the *niche* where they are confined.^[^ ^18^ ^]^ For this reason, the osmolality and pH of the hydrogel are controlled to emulate the physiological conditions. Made essentially of water (99.9%), nanocellulose hydrogel has no osmolality (0 mOsmol kg^−1^). Glycine (250 mm), a non‐polar amino acid, is dissolved in the hydrogel to increase its osmolality to 295 mOsmol kg^−1^ (Figure 1h), matching Matrigel (290 mOsmol kg^−1^). This strategy not only preserves the hydrogel colloidal stability before the ionic crosslinking takes place, but also conserves cell viability until the culture media is added to the wells. HEPES (25 mm), a widely used buffering agent in cell culture, is also added to buffer the matrix pH to the physiological range. A highly hypotonic material induces massive apoptosis within 24 h causing crypts to turn into an aggregate of debris. In addition, an acidic hydrogel also impacts the survival and growth of crypts encapsulated within the matrix (Figure S5, Supporting Information). In this study, the combination of glycine and HEPES is referred to as GLY. The preliminary test of neat, GLY‐ and RGD‐GLY hydrogels as matrices for organoid growth is shown in Figure 1i. *Mus musculus* small intestinal organoids were adopted as the biological model and initially established in Matrigel. After passaging, mouse crypts embedded in neat hydrogel turn into an agglomerated cluster of debris within 2 days. Once the osmolality of the matrix is balanced by GLY, small intestinal crypts progress into cystic organoids. Organoids are formed only in the presence of RGD‐GLY hydrogel, showing morphology and size similar to the crypts established in Matrigel.

**Figure 2:**
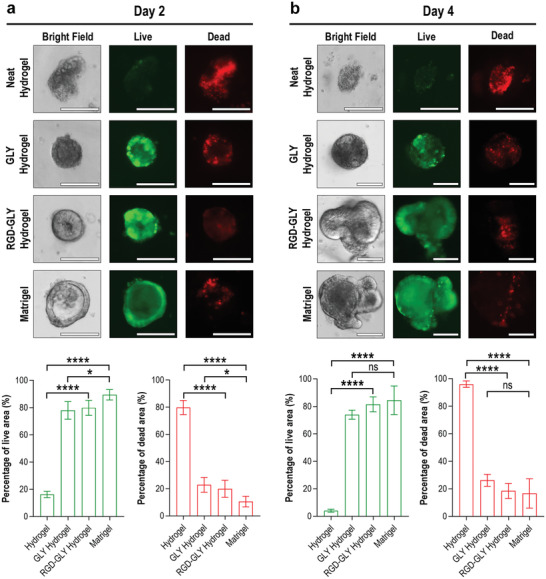
Small intestinal organoid viability. A) Crypts were seeded in neat, GLY, and RGD‐GLY nanocellulose hydrogel. After 2 days, a major apoptotic area is detected in the samples cultured in the neat hydrogel. Once GLY is added, cystic organoids are formed, predominantly composed of living cells. However, morphology and topography strongly differ from the control (Matrigel). Crypts seeded in RGD‐GLY hydrogel form cystic organoids, presenting a development similar to those in Matrigel with 80% viability. B) After 4 days, RGD‐GLY hydrogel induces progression to budding organoids. In the absence of RGD peptides, the organoids remain cystic, growing in size only. Scale bars: 100 µm. Results shown represent independent experiments performed in triplicates (*n* = 3, error bars = SD). * = *p* < 0.05. **** = *p* < 0.0001. ns = non‐significant.

In order to assess the biocompatibility of engineered hydrogels, small intestinal crypts are embedded in different formulations of the material and cultured for 4 days. On the second and fourth day, calcein AM staining was used to identify the viable cells and propidium iodide to reveal the dead cells. Crypts encapsulated in the neat nanocellulose hydrogel exhibit a high level of apoptosis, with on average 80% of each unit containing dead cells, with only 20% viable cells after 2 days. Once the gel osmolality is balanced and the matrix is buffered by GLY, the viable area within cystic organoids is increased to 78% and the dead area reaches 22%. Grafting RGD peptides onto the cellulose nanofibers does not affect overall viability, preserving 80% and 20% of living and dead areas, respectively. Organoids embedded in Matrigel present viability of 88% and an apoptotic region of 18% after being cultivated for 48 h (**Figure** **2**a). On the fourth day of culture, crypts in the neat hydrogel continue as a cluster of apoptotic debris, representing 97% of its area. Organoids encapsulated within the GLY hydrogel sustain 75% of viability with the remaining 25% of organoid area containing apoptotic cells. RGD‐GLY hydrogel supports 81% viability, indicating the maintenance of cells in small intestinal organoids to be similar to those seeded in the EHS matrix (80% living and 20% dead areas) (Figure 2b).

**Figure 3:**
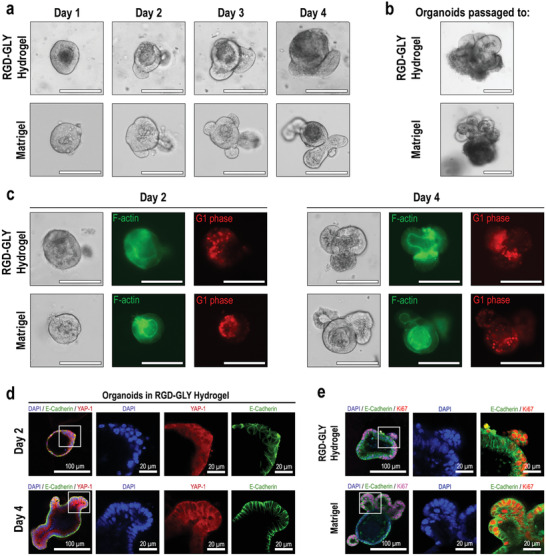
Characterization of organoids grown in RGD‐GLY hydrogel. A) Crypts cultured in RGD‐GLY nanocellulose hydrogel and Matrigel acquired the cystic phase within 1 day and form organoids after 3 days. B) Organoids cultured in RGD‐GLY hydrogel which have been passaged to RGD‐GLY and returned to Matrigel, exhibit similar morphology after 7 days. C) F‐Actin filaments and cells‐RFP in G1 phase are observed in FUCCI organoids cultured in RGD‐GLY hydrogel and Matrigel. D) Cystic and budded organoids show the subcellular localization of YAP‐1. E) Proliferative cells stained for Ki67 are observed in the organoids embedded in RGD‐GLY hydrogel and Matrigel. Scale bars: 100 µm. Results shown represent independent experiments performed in triplicates (*n* = 3).

The ability of nanocellulose hydrogels to establish organoids from freshly isolated mouse small intestine crypts was assessed. Whilst organoids are easily established in Matrigel, dissected crypts directly seeded in RGD‐GLY hydrogel do not progress into organoids. However, when the hydrogel is supplemented with a minor volume of Matrigel—20% (v/v)—, organoids are established from fresh intestinal crypts (Figure S6, Supporting Information). The combination of nanocellulose and Matrigel at this condition does not alter the stiffness of the matrix (Figure S7, Supporting Information). Interestingly, organoids could also be formed from single cells seeded in RGD‐GLY hydrogel, although the growth rate is considerably slower than those embedded in Matrigel (Figure S8, Supporting Information). Once established and passaged, the growth of organoids embedded into RGD‐GLY hydrogel (absent of any volume of Matrigel) is tracked over four days and compared to those within the EHS matrix. On day 1, small intestinal crypts embedded within both materials acquire spherical morphology with a lumen characteristic of the cystic phase. On the second day, the spheres become asymmetric with the initiation of budding. The following day, organoids in RGD‐GLY hydrogel and Matrigel contain fully formed budding crypts, requiring passage on the fourth day (**Figure** **3**a). Interestingly, all the organoids cultured in GLY hydrogel remain in their cystic phase without forming buds even after 7 days (Figure S9, Supporting Information). The presence of adhesive sites plays a key role in the process of organoid development, and removal is known to inhibit the formation of buds. Indeed, the lack of integrin beta‐1 ligand induces the reversion of polarization, with the external exposure of the apical layer and internalization of the basal region.^[^ ^19^ ^]^ Despite the disruption of this process in GLY hydrogels, cells are still viable, and organoids grow in size. In contrast, RGD‐GLY hydrogel promotes crypts to form cystic organoids that evolve to budding units in 2 or 3 days and require to be passaged after 4 days. However, some cystic organoids are still present in RGD‐GLY hydrogel (Figure S10, Supporting Information). The organoids cultured in the RGD‐GLY hydrogel were passaged every 4 days for 2 weeks, preserving the expected development and morphology (Figure S11, Supporting Information). Organoids passaged from nanocellulose back to Matrigel also present the usual morphology and normal growth for 7 days (Figure 3b). These results indicate that both the nanocellulose and the animal‐based matrix are interchangeable, whilst engineered nanocellulose seems not to interrupt the self‐renewing ability of the stem cell population and subsequent self‐organizing processes present in the organoids.

We further examined cytoskeletal and proliferative cell markers to characterize organoid growth in nanocellulose hydrogels. The development of organoids within the nanocellulose hydrogel and Matrigel was analyzed by culturing organoids derived from fluorescence ubiquitination cell‐cycle indicator (FUCCI) mice in both matrices.^[^ ^20^ ^]^ F‐actin filaments are observed by the presence of LifeAct‐green fluorescent protein (GFP) in FUCCI organoids. Prominent apical staining of the well‐known network of F‐actin, which support microvilli, was observed indicating the preservation of cytoskeleton integrity and cellular polarity in both cystic and budded phases. A similar proportion of red fluorescent protein (RFP)‐labeled cells in G1 phase of the cell cycle was also observed in organoids cultured in nanocellulose for 2 and 4 days (Figure 3c). Proliferative cells are found in small intestinal organoids revealed by staining for cells expressing the nuclear protein Ki67, after 3 days of culture in nanocellulose hydrogel and Matrigel (Figure 3e). Metabolic activity is increased by 75% during the first 2 days of culture in the hydrogel and remains stable on the fourth day (Figure S12, Supporting Information). Interestingly, yes‐associated protein 1 (YAP‐1), a mechanosensing effector of the Hippo signaling pathway required for initiation of intestinal organoid budding, is shown to be inactive in both cystic and budded organoids (Figure 3d).^[^ ^11^, ^21^ ^]^ Recently, based on organoids formed from single stem cells, Serra et al. clarified that YAP‐1 is active and localized within the nuclei during the first 24 h of culture, and then re‐localized to the cytosol once inactivated over time.^[^ ^21^ ^]^ However, since the organoids assessed here were not formed from single cells, the transient activation of YAP could not be detected. This suggests that the organoids could have been seeded after breaking of the initial symmetry has occurred in the early stages. Overall, the RGD‐GLY hydrogel provides the biological conditions required for organoids to develop from crypts to cystic organoids which progress to mature organoids containing crypt domains.

**Figure 4:**
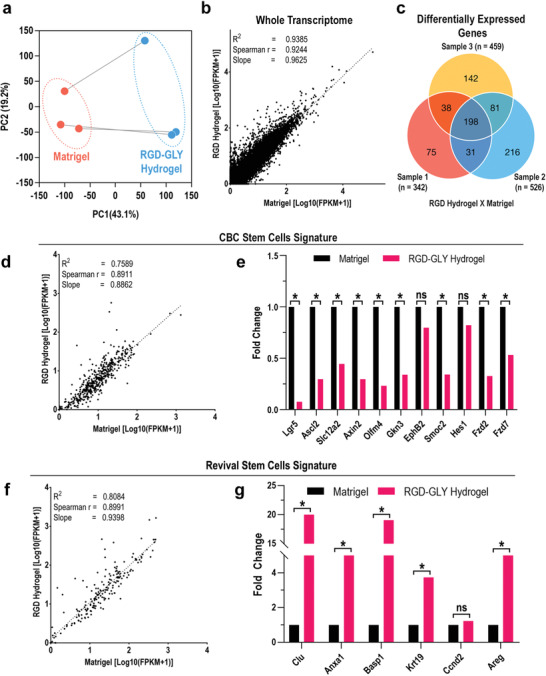
Transcriptomic analysis of organoids cultured in RGD‐GLY hydrogel. A) Principal component analysis (PCA) shows the effect size of variances in the mRNA expression of small intestinal organoids cultured in Matrigel and RGD‐GLY hydrogel. B) Correlation analysis comparing the whole transcriptome shows a strong correlation. C) Veen diagram shows 198 genes to be commonly differentially expressed in three organoid samples. D) Organoids cultured in Matrigel and RGD‐GLY hydrogel present a high correlation between crypt base columnar (CBC) stem cell markers. E) Several CBC‐related genes are downregulated in the hydrogel compared to Matrigel. F) Expression of revival stem cell markers also preserve high correlation between the organoids growing in both materials. G) An increasing of up to 20‐fold is found in genes like *Clu* and *Basp1*. Results shown represent independent experiments performed in triplicates (*n* = 3). * = Fold change > 2, FDR and *p* < 0.05. ns = non‐significant.

To further assess the characteristics of organoids cultured in RGD‐GLY hydrogel compared to Matrigel, comparative transcriptomic analysis was performed. RNA sequencing reveals that small intestinal organoids embedded in nanocellulose exhibit some differences in expression of specific groups of genes when compared to those in Matrigel. Biological variations are also detected between organoids derived from three different mice, and cultured in each material (**Figure** **4**a). Overall, a good correlation in the whole transcriptome is observed, with the coefficient of determination as high as 0.93 (Figure 4b). One hundred ninety eight genes are identified to be significantly differentially expressed in organoids growing in nanocellulose with fold change ≥ 2 (FDR and *p* ≤ 0.05) (Figure 4c), notably those related to receptor ligand activity and calcium‐dependent binding (Figure S13, Supporting Information). This event is expected and understandable, considering the ionic crosslinking method explored to provide the mechanical properties of the hydrogel. Interestingly, the expression of crypt base columnar (CBC) stem cell markers, such as *Lgr5* and *Ascl2*, decreases in RGD‐GLY hydrogel (Figure 4e), whilst those related to revival stem cells present up to a 20‐fold increase (Figure 4g).^[^ ^22^ ^]^ CBC stem cells refer to a population of cells responsible for the self‐renewability of the intestinal epithelium.^[^ ^23^ ^]^ Recently, a population of quiescent stem cells—named revival stem cells—was characterized on its ability to drive the regeneration of damaged intestinal tissue and to reconstitute the eventual loss of CBC stem cells.^[^ ^24^ ^]^ Both populations are of critical importance in reliable intestinal models. Despite these variations, the Spearman's correlation coefficient for CBC and revival stem cells genes remains as high as 0.89 (Figures 4d,f). The expression of proliferative markers is downregulated, in contrast to the YAP signaling and differentiation genes which appear to be enhanced (Figure S14, Supporting Information). Indeed, markers related to Paneth (*Oit3* and *Ang4*), tuft (*Trpm5* and *Gfi1b*) and goblet cells (*Muc2* and *Tff3*) as well as genes associated to enteroendocrine cells (*Tph1* and *Reg4*) and enterocytes (*Alpi* and *Apoa1*) are found in higher levels for organoids in the cellulosic matrix (Figure S15, Supporting Information). These differences can be justified by the high number of growth factors and other biomolecules existing in Matrigel but absent in RGD‐GLY hydrogel.^[^ ^5^, ^7^ ^]^ Our simplified system is essentially made of 99.9% of water and only 0.1% solids content, functionalized with a single cell adhesive peptide. In this context, the ability to supplement the hydrogel with ECM‐derived elements is welcome, allowing the design of a controlled microenvironment for specific needs. Overall, the culture of small intestinal organoids in standard RGD‐GLY hydrogel may be a good model that supports studies focused on differentiated cell types and the process of regeneration following injury.

**Figure 5:**
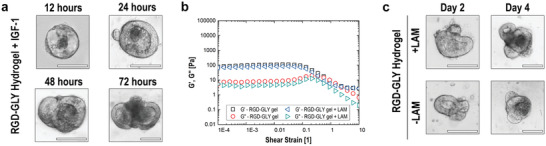
Effect of additives on nanocellulose hydrogel. A) Early‐stage organoids are observed in RGD‐GLY hydrogel supplement with IGF‐1 in less than 1 day. B) The addition of laminin‐1 to RGD‐GLY hydrogel does not affect the rheology of the material. C) Organoids cultured in the presence and absence of laminin‐1 present similar development. Scale bars: 100 µm. Results shown represent independent experiments performed in triplicates (*n* = 3).

As a proof‐of‐concept of the tunability of nanocellulose hydrogel, this matrix was combined to insulin‐like growth factor‐1 (IGF‐1) and laminin‐1; these are two of the elements present in Matrigel. IGF‐1 has the ability to induce the crypt expansion by activation of intestinal stem cells.^[^ ^25^ ^]^ Laminin‐1 is the major protein found in the EHS matrix and forms a network associated with collagen, fibronectin and other proteins.^[^ ^26^ ^]^ More than reinforcing the mechanical properties, laminin also promotes epithelial cell adhesion and differentiation.^[^ ^27^ ^]^ The concentration of IGF‐1 in the EHS matrix ranges between 1.7 to 4.7 ng mL^−1^, whilst laminin makes up to 60% of the protein content in Matrigel. The addition of IGF‐1 at its minimal concentration to the hydrogel accelerates the expansion of early‐stage cystic organoids in less than 24 h. Within 3 days of culture, organoids in IGF‐containing hydrogel expand substantially in size (**Figure** **5**a). In contrast, organoids growing in IGF‐free hydrogel require at least 5 days to achieve similar dimensions (Figure S16, Supporting Information). This indicates that IGF‐1 might contribute to the development of the organoids seen in the EHS matrix. On the other hand, the addition of laminin‐1 (0.5 mg mL^−1^) has minimum effects on the nanocellulose rheological properties, preserving the storage and loss moduli at 94 and 6 Pa, respectively (Figure 5b). Organoids grown in hydrogels containing this protein present a similar morphology to those cultured in absence of laminin‐1 (Figure 5c). Previous works reported that enriched laminin‐materials (up to 3 mg mL^−1^) benefited the formation and growth of organoids.^[^ ^11^, ^13^ ^]^ However, the success of these gels might simply be due to the generous amount of laminin, also extracted from mouse sarcoma. In contrast, Rezakhani et al. recently showed that laminin‐1 in gels at concentrations as low as 0.3 mg mL^−1^ can also improve organoid growth, whilst laminin‐free hydrogels were found to support organoid derivation.^[^ ^28^ ^]^ Yavitt et al. also demonstrated that hydrogels without laminin were restricted to expand intestinal stem cell colonies, but could not form organoids.^[^ ^29^ ^]^ In this study, the effect of a higher concentration of laminin was not evaluated; this was to maintain functionalized nanocellulose fibers as the predominant element in a fully defined plant‐based hydrogel. Inspired by these outcomes, the addition of the ECM‐derived factors can be further explored to achieve the conditions required to sustain different organoids derived from other organs.

## Conclusion

To the best of our knowledge, we introduce the first engineered plant‐based nanocellulose hydrogel as a very low‐cost but performant material for organoid growth. Organoids represent a robust model for key applications in biomedicine including drug screening and disease modelling. The EHS matrix has become the standard material for organoid culture and is widely used. However, Matrigel and similar matrices remain expensive, biochemically variable and undefined. These are major obstacles for fundamental research studies and the translation of organoids to clinics. Alternative matrices able to sustain organoid systems are required to drastically reduce costs and to eliminate the liability from unknown biomolecules. These materials must be compliant with good manufacturing practices (GMP) procedures required for reproducible and accredited pre‐clinical tests. Synthetic ECM‐like structures allow organoids to be cultivated for further use as tissue replacement in regenerative medicine. Our hydrogel has similar mechanical properties to those of the EHS matrix. Easy to functionalize, cellulose nanofibers are grafted with RGD peptides, inducing small intestinal organoids formation and growth. Both matrices are interchangeable, showing the presence of proliferative cells and regular cell cycle. In this study, mouse small intestinal organoids were adopted as biological model. Despite the positive outcomes reported, nanocellulose is limited by not establishing organoids from dissected tissues. In addition, other types of organoids, including those derived from human tissues, must be individually evaluated to determine the ideal mechanical and biochemical conditions of specific cultures. Overall, nanocellulose hydrogel stands as a promising material for its tunability and compatibility with ECM‐components. Indeed, based on recent reports, the specific combination of nanocellulose and laminin may support the long‐term culture of organoids, allowing multiple passages. Engineered nanocellulose hydrogel represents a performant and sustainable alternative for the growth of organoids, contributing to significantly reducing the costs in studies against diseases of global concern such as cancer.

## Experimental Section

##### Synthesis of Nanocellulose Hydrogel

A 4 wt% never dried bleached Eucalyptus Kraft (BEK) pulp suspension (Australian Paper, Maryvale, Australia) containing proportional amounts of TEMPO (Sigma‐Aldrich 214000) and NaBr (Sigma‐Aldrich 71329) was prepared according to Saito et al.^[^ ^30^ ^]^ To initiate the oxidation, 12 w/v% NaClO (Sigma‐Aldrich 425044) (pH adjusted to 10) was added drop‐wise at a ratio of 5 mmol NaClO g^−1^ fiber. The reaction was maintained at pH 10 by adding 0.5 m NaOH. The oxidation process was proceeded until reaction termination—corresponding to the stabilization of pH. Oxidized fibers were recovered and washed through filtration with a Buchner funnel and stored refrigerated (4 °C). The oxidized pulp was dispersed in deionized water to achieve a concentration of 0.1 wt% and converted into gels via mechanical fibrillation through a high‐pressure homogeniser (GEA Niro Soavi Homogenizer Panda) at 1000 bar. The carboxylate group content was determined by conductometric titration as previously reported.^[^ ^30^, ^31^ ^]^ Freeze‐dried oxidized pulp samples (≈30 mg) were suspended into 40 mL deionised water. Then, 40 µL 1% NaCl was added to the suspended sample. The pH of the suspended sample was adjusted between 2.5 and 3 prior to titration with 0.01 m NaOH using a Mettler Toledo T5 titrator. The conductivity of the sample was monitored throughout the titration progress. The carboxyl group content CC (mmol COO^−^Na^+^ g^−1^ fiber) was determined as:

(1) $$\mathrm{CC}=\frac{c\left(V_{2}-V_{1}\right)}{w}\times 1000$$

Where *V*_1_ and *V*_2_ are the amount of titrant (start and end of conductivity titration curve) required to neutralize the carboxylic groups (in L), c is the NaOH concentration (mol L^−1^), and *w* is the sample weight (g).

##### Hydrogel Functionalization

Cellulose nanofibers were functionalized with RGD peptides via EDC/NHS coupling. EDC (Sigma‐Aldrich E6383) and NHS (Sigma‐Aldrich 130672) were dissolved in 2‐(*N*‐morpholino)ethanesulfonic acid (MES, Sigma Aldrich M0164) buffer containing NaCl (Merk 1064040500) 0.9% at pH 6. To activate the carboxylate groups, equimolar solutions of EDC and NHS were added to the gel in a mole ratio 1:1:1 to the existing carboxylate concentration and stirred 15 min at room temperature. A volume of (GRGDSPC) RGD peptides (GenScript) (final concentration = 2 mm) was added to the hydrogel and stirred for 2 h at room temperature. The pH of the hydrogel was adjusted to 7 by adding NaOH (Sigma‐Aldrich S8045) (1 m) measured by pH strips. Glycine (Sigma‐Aldrich 50046) (250 mm) and 4‐(2‐hydroxyethyl)‐1‐piperazineethanesulfonic acid (HEPES, Sigma‐Aldrich H7523) (25 mm) were dissolved in the hydrogel. A volume of 100 µL was used to measure the osmolality in a K‐7400S osmometer (Knauer). The pH was adjusted to 7 by addition of NaOH (1 m), measured with pH strips. The hydrogel was sterilized under ultraviolet radiation exposure during 20 min prior to use. For functionalization, hydrogels were mixed with IGF‐1 and laminin‐1, at 5 ng mL^−1^ and 0.5 mg mL^−1^ final concentration, respectively.

##### Rheology

Nanocellulose hydrogels and Matrigel were characterized by rheology using an Anton Paar MCR302 rheometer with a parallel plate. All testing was performed at 37 °C and a solvent trap was used to ensure temperature stability. Two types of rheological tests were performed: viscosity measurements and oscillatory strain sweep. Viscosity measurements were performed between shear rates of 0.5 to 100 s^−1^. Oscillatory strain sweep was measured between 0.01% and 100% strain at constant 1 Hz frequency. A volume of 1 mL of nanocellulose hydrogels was crosslinked with 100 µL CaCl_2_ (100 mm) and incubated at room temperature for 5 min. A volume of 1 mL of Matrigel was incubated at 37 °C, 5% CO_2_ for 15 min. Samples were covered with organoid culture media and incubated at 37 °C for 3 h. After incubation, the culture media was removed and the hydrogels or Matrigel transferred to the rheometer surface for measurements. For stability test, RGD‐GLY hydrogel was prepared in a similar manner and then incubated at 37 °C, 5% CO_2_ during 1 to 4 days before measurement. All measurements were performed in triplicates to ensure repeatability.

##### Small‐Angle Neutron Scattering

Small‐angle neutron scattering (SANS) experiments were made at the Time‐of‐Flight BILBY beamline at the Australian Nuclear Science and Technology Organization (ANSTO), NSW, Australia. The D_2_O‐hydrated samples were placed in the demountable cells of wall thickness 2 mm. For measurements, neutrons of wavelength of 6 Å was selected by neutron‐velocity selector. Two sample to detector lengths were applied which cover the Q‐range from 0.00254 to 0.31Å^−1^. The raw collected data was reduced by the Mantid software with the BILBY package. During data reduction, the background of the D_2_O was subtracted from the hydrated samples. The reduced data was normalized to the absolute scattering values by the pre‐calibrated scattering curve of D_2_O. Data analysis was performed by SASview.

##### Small Intestinal Organoids Culture

All animal procedures were approved by the Monash Animal Ethics Research Platform ethics committee in strict accordance with good animal practice as defined by the National Health and Medical Research Council (Australia) Code of Practice for the Care and Use of Animals for Experimental Purposes. Mice (*M. musculus*) were culled by cervical dislocation. The small intestine tube was dissected and flushed with PBS to remove feces. Small intestinal tissues were opened longitudinally, scraped with a glass coverslip to remove villi, cut into 5‐mm pieces and washed with PBS five times to remove unattached epithelial fragments, mucus and feces. Following incubation for 30 min at 4 °C in 3 mm EDTA‐PBS solution, intestinal crypts were released from small intestine tissue fragments by mechanically pipetting them with a 10 mL pipette in PBS and repeating this step three times. Isolated intestinal crypts were strained (70‐µm cell strainer, Falcon) and pelleted by centrifugation three times at 1500 rpm for 2 min at 4 °C. The supernatant was discarded and the pellet containing the crypts was re‐suspended in Growth Factor Reduced (GFR) Matrigel (Corning 356 231), nanocellulose hydrogels or in hydrogels supplemented with Matrigel (20% v/v). A volume of Matrigel containing crypts (50 µL) was seeded into a 24‐well plate (Nunc) and incubated for 15 min at 37 °C until solidified. For nanocellulose hydrogels, the 24‐well plate was previously coated with 5 µL of calcium chloride (100 mm), then followed by the seeding of 50 µL of hydrogels containing intestinal crypts. Hydrogels were incubated for 5 min at room temperature. Each well containing each matrix received 500 µL of crypt culture media (DMEM/F12 (Gibco), B27 (Gibco), Glutamax (Gibco), N2 (Gibco), 10 mm HEPES (Gibco), Penicillin‐Streptomycin (Gibco), 0.5 µg mL^−1^ Amphotericin B (Gibco), 50 ng mL^−1^ EGF (Peprotech), 2% Noggin conditioned media, and 10% R‐spondin 1 conditioned media). Crypt culture media was replaced every 2 days, and organoids were passaged every 3–4 days. For passaging, Matrigel or nanocellulose hydrogels were mechanically disrupted and the organoids recovered by centrifugation (3 min at 1500 rpm). Residual volumes of Matrigel or hydrogel were removed, and the organoids resuspended in DMEM/F12 media followed by mechanical disruption or dissociation achieved by manual pipetting for at least 30×. For single cell experiments, the organoids were resuspended in TrypLE Express (Invitrogen, 12604021) and incubated 5 min at 37 °C. Crypts or cells were washed with 5 mL of DMEM/F12 media during 3 min at 1500 rpm and resuspended in Matrigel or nanocellulose hydrogels for seeding as previously described.

##### Viability Test

Small intestine organoids encapsulated in the hydrogels or Matrigel were cultured during 2 or 4 days and stained with Calcein‐AM (Sigma‐Aldrich C1359) and Propidium Iodide (Sigma‐Aldrich P4170). The crypt culture media was replaced by 500 µL of DMEM/F12 media containing 1 µm calcein‐AM and 3 µm propidium iodide. The plate was incubated at 37 °C 5% CO_2_ during 30 min in absence of light. Organoids were imaged under EVOS XL Core microscope. Quantification of living and dead area was performed by image processing using FIJI software and data analysis with OriginPro 9.

##### Metabolic Activity

Organoids in Matrigel or RGD‐GLY hydrogel had their metabolic activity measured by the Prestoblue (Invitrogen A13261) assay. Prestoblue solution was diluted in DMEM/F12 media (10% v/v) and incubated at 37 °C for 5 min; 200 µL was added to each well (matrix and organoids and matrices only). Samples were transferred to the incubator (37 °C, 5% CO_2_) for 40 min. Fluorescence was measured by a Pherastar FSX plate reader (540 nm excitation; 590 nm emission). Values from days 2 and 4 were normalized to those from day 1 (100%).

##### Organoid Characterization

Small intestinal organoids were cultured in hydrogel or Matrigel for 4 days and immunostained for Ki67, E‐cadherin and YAP‐1. Organoids were washed twice times with 500 µL of PBS at room temperature. Organoids were washed three times with 500 µL of 100 mm glycine in PBS for 10 min at room temperature. Organoids were incubated overnight with 500 µL immunofluorescent buffer (0.1% Bovine Serum Albumin (Sigma‐Aldrich A3311), 0.2% Triton X‐100 (Sigma T9284), 0.05% Tween 20 (Sigma P9416), 10% horse serum, PBS). Organoids were incubated overnight with primary antibodies (mouse anti‐Ki67 (Dako M7248), rabbit anti‐E cadherin (Abcam Ab53033) and mouse anti‐YAP‐1 (Santa Cruz 101 199)) 1:500 diluted in 1% BSA in PBS at 4 °C. Organoids were washed three times with IF buffer, once in the morning and once at lunch time at room temperature, and the last time over night at 4 °C. Organoids were incubated in secondary antibody (goat anti‐mouse IgG conjugated with Alexa Fluor 488, Invitrogen A28175) 1:2000 diluted in 1% BSA in PBS (protected from light) at 4 °C overnight. Organoids were counterstained with DAPI (Invitrogen D1306, 1:5000 dilution in 1% BSA in PBS) for 30 min at room temperature. Organoids were washed PBS for 10 min × 3 at room temperature. FUCCI organoids expressing f‐actin‐GFP and cells‐RFP were kindly provided by Dr. Thierry Jarde, having been established according to Sakaue‐Sawano et al. and Riedl et al.^[^ ^20^ ^]^ Organoids were imaged under Leica AF6000LX and EVOS XL Core microscopes.

##### RNA Isolation and Sequencing

Organoids isolated from three different mice were cultivated for 4 days in both Matrigel and hydrogel. The complete media was removed and wells washed three times with filtered PBS. A volume of 1 mL TRIZol (Invitrogen) was used to dissolved Matrigel and hydrogels and release the cellular content. Chloroform (200 µL) was added to TRIzol and tubes vigorously mixed for 15 s. After 3 min incubation at room temperature, samples were centrifuged at 14 000 rpm for 15 min at 4 °C. The aqueous phase was transferred to empty tubes and combined to 500 µL of isopropanol. Samples were incubated at room temperature for 15 min and then centrifuged at 14 000 rpm for 10 min at 4 °C. The supernatant was removed and the pellet washed with cold ethanol (75% v/v). Sample were mixed and centrifuged as before. The supernatant was removed and pellet was air dried for 5 min at room temperature. A volume of 15 µL of RNase‐free water was added to the tubes. Sample were dried in RNA stabilization tubes (GENEWIZ) by centrifugal vacuum concentrator (Eppendorf) for 30 min at 1500 rpm in 30 °C. RNA sequencing was performed and analyzed by GENEWIZ. Briefly, total RNA of each sample was quantified and qualified by Agilent 2100 Bioanalyzer (Agilent Technologies). 1 µg total RNA with RIN value above 6.5 was used for library preparation by poly(A) mRNA Magnetic Isolation (NEBNext) for poly(A) mRNA isolation, then priming with First Strand Synthesis Reaction Buffer and Random Primers followed by cDNA synthesis using ProtoScript II Reverse Transcriptase and Second Strand Synthesis Enzyme Mix. After T‐A ligation, size selection (≈420 base pairs) and PCR amplification, the PCR products were cleaned up and quantified by Qubit3.0 Fluorometer (Invitrogen). Multiplexed RNA sequencing was performed on Illumina HiSeq instrument using a 2 × 150 bp paired‐end configuration which revealed more than 50 m reads. Quality control and assessment were performed using Cutadapt (V1.9.1) and FastQC (V0.10.1) respectively.^[^ ^32^ ^]^ Sequence reads were then aligned to *M. musculu*s genome (ENSEMBL:GRCm38.97) using Hisat2 (v2.0.1).^[^ ^33^ ^]^ Gene expression level was determined using HTSeq (V0.6.1).^[^ ^34^ ^]^ Differential gene expression of mouse small intestinal organoids grown in Hydrogel relative to in Matrigel was calculated using the Bioconductor package DESeq2 (V1.6.3)^[^ ^35^ ^]^ and reported in fold change.

##### Statistical Analysis

The statistically significant differences in live and dead areas of organoids were assessed by one‐way analysis of variance (ANOVA) with Tukey's multiple comparisons test in Graphpad Prism 8. For RNAseq, genes with fold change ≥ 2 with False Discovery Rate (FDR) and adjusted *p* value < 0.05 were considered significantly differentially expressed. Principal component analysis (PCA) was performed using R to visualize sample‐to‐sample distances. Gene ontology (biological process) analyses was performed using Metascape.^[^ ^36^ ^]^ Correlation analyses were performed using liner regression and Spearman's Rank‐Order in Prism 7 to compare the expression profiles of whole transcriptome and cell‐types specific marker genes between organoid from different matrices.

## Conflict of Interest

The authors declare no conflict of interest.

## Author Contributions

R.C. designed and performed all the experiments and wrote the manuscript. G.K., D.J.M., W.H.C. were involved in performing and analyzing the RNAseq and immunostaining of organoids. V.S.R. analyzed the data from small angle neutron scattering experiments. J.R., H.E.A., and G.G. reviewed the manuscript and provided scientific vision and consultation along the study.

## Supporting information

Supporting Information
