# Supplementary material for: Engineered Plant‐Based Nanocellulose Hydrogel for Small Intestinal Organoid Growth
Source: Adv Sci (Weinh). 2020 Nov 20;8(1):2002135. doi: 10.1002/advs.202002135 (PMC7788499; doi:10.1002/advs.202002135)
Supplement: Supplementary file 1 — Supporting Information [file ADVS-8-2002135-s001.pdf]

© 2020 Wiley-VCH GmbH

ADVANCED SCIENCE Open Access

Supporting Information

for *Adv. Sci.*, DOI: 10.1002/advs.202002135

Engineered Plant-based Nanocellulose Hydrogel for Small Intestinal Organoid Growth

*Rodrigo Curvello, Genevieve Kerr, Diana J. Micati, Wing Hei Chan, Vikram S. Raghuwanshi, Joseph Rosenbluh, Helen E. Abud and Gil Garnier**

Engineered Plant-based Nanocellulose Hydrogel for Small Intestinal Organoid Growth

Supplementary Information

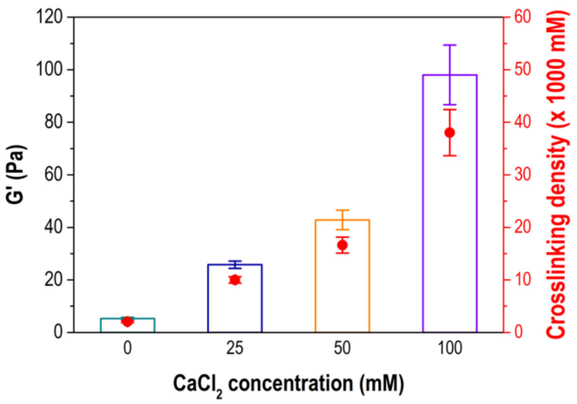

Figure S1. Evaluation of stiffness in crosslinked nanocellulose hydrogel. Hydrogels were crosslinked at different concentrations of $CaCl_2$. Stiffness is referred as the average of the storage modulus in the linear viscoelastic region (LVR). Results shown represent independent experiments performed in triplicates (n=3, error bars = SD).

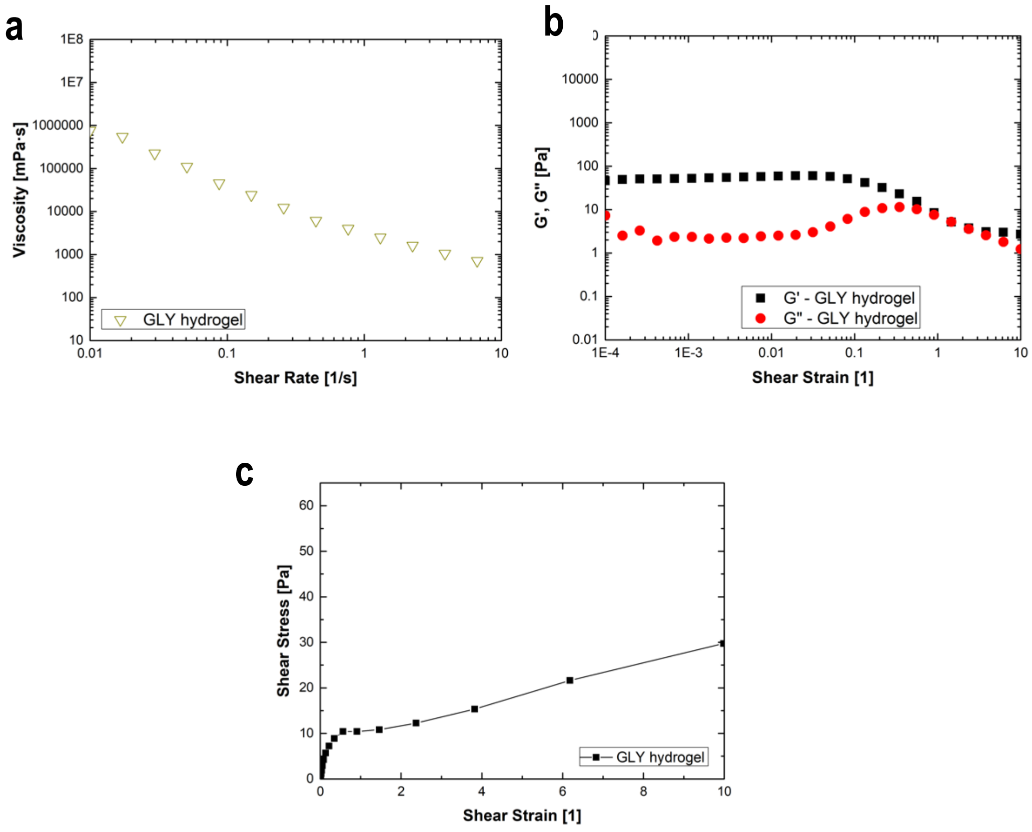

Figure S2. GLY hydrogel characterization. (A) GLY hydrogel presents shear-thinning behavior. **(B)** Storage (G') and loss (G") moduli of GLY hydrogel also match the viscoelastic rheology of Matrigel. **(C)** GLY hydrogel flows under a shear of 10 Pa. Results shown represent independent experiments performed in triplicates (n=3).

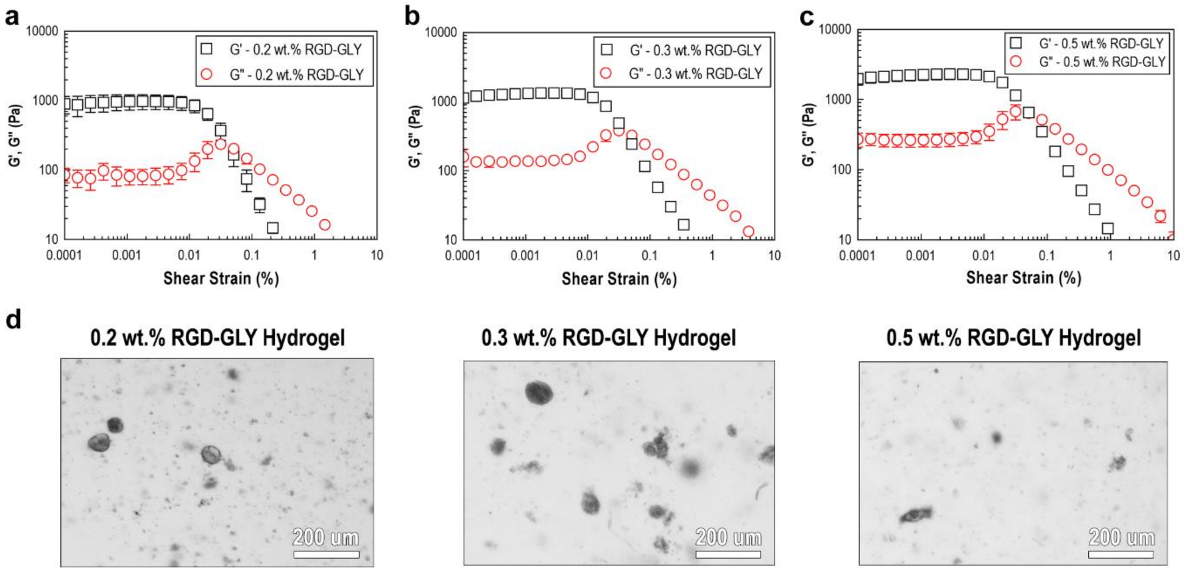

Figure S3: Nanocellulose hydrogel overview and characterization. Storage and loss moduli of RGD-GLY hydrogel at **(A)** 0.2, **(B)** 0.3 and **(C)** 0.5 wt.% solids content are stiffer than Matrigel and **(D)** do not induce intestinal organoid growth. Results shown represent independent experiments performed in triplicates (n=3, error bars = SD).

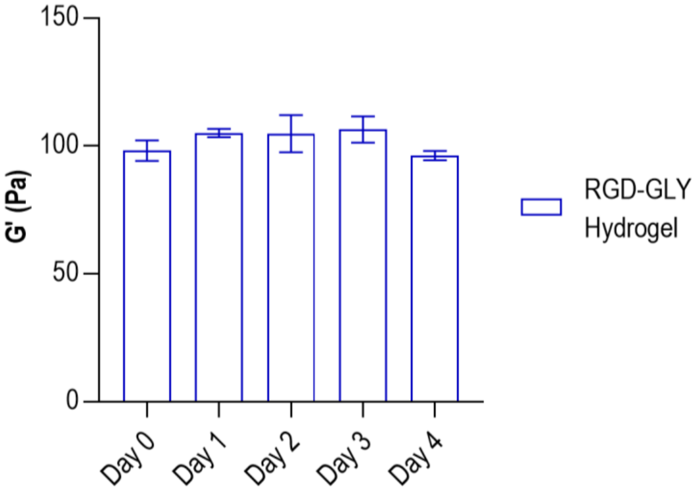

Figure S4: Stability test. RGD-GLY hydrogels do not degrade over time, retaining the initial storage modulus (G') over time. Results shown represent independent experiments performed in triplicates (n=3, error bars = SD).

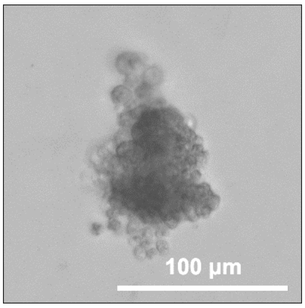

Figure S5: Small intestinal organoid in acidic matrix. Small intestinal crypt was cultured in RGD-GLY hydrogel at pH 4, losing its integrity after 24 hours. Results shown represent independent experiments performed in triplicates (n=3).

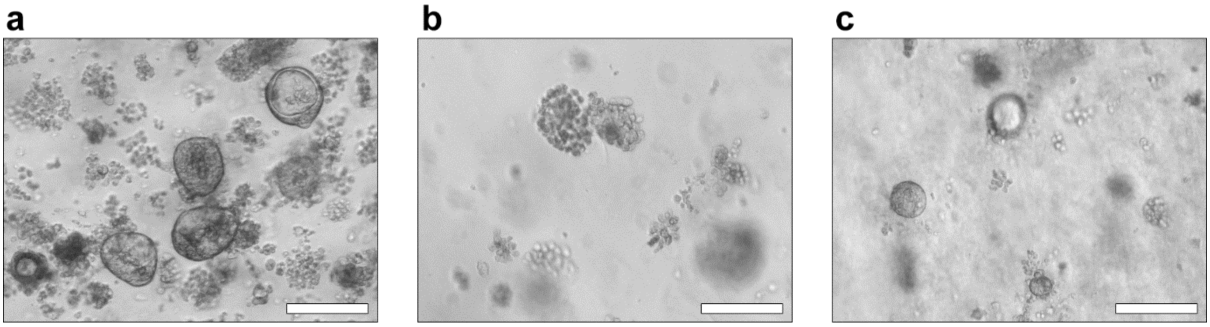

Figure S6: Establishing organoids. Fresh mouse small intestinal crypts seeded into **(A)** Matrigel and **(B)** RGD-GLY hydrogel. Organoids are formed in Matrigel but not in RGD-GLY hydrogel. **(C)** Small intestinal organoids are established in hydrogels supplemented with Matrigel – 20% (v/v). Scale bars: 100 µm.

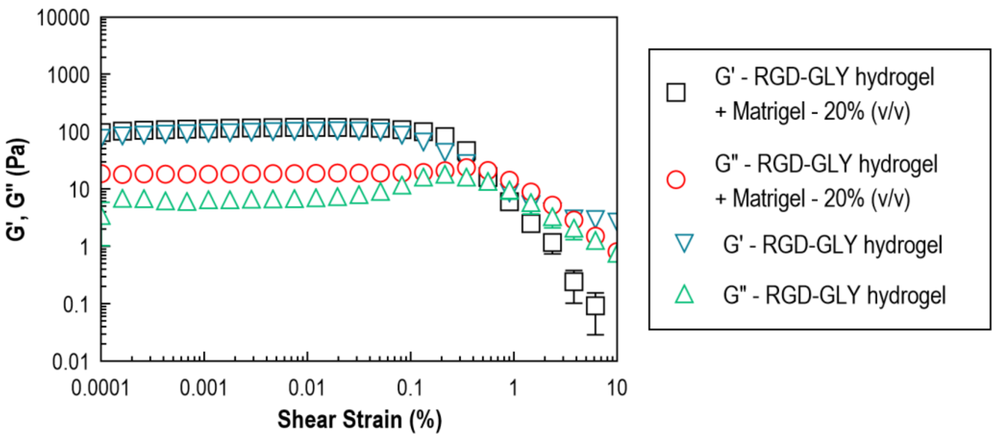

Figure S7: Nanocellulose hydrogel-Matrigel characterization. Storage and loss moduli of RGD-GLY hydrogel in absence of and supplemented with Matrigel - 20% (v/v). Results shown represent independent experiments performed in triplicates (n=3, error bars = SD).

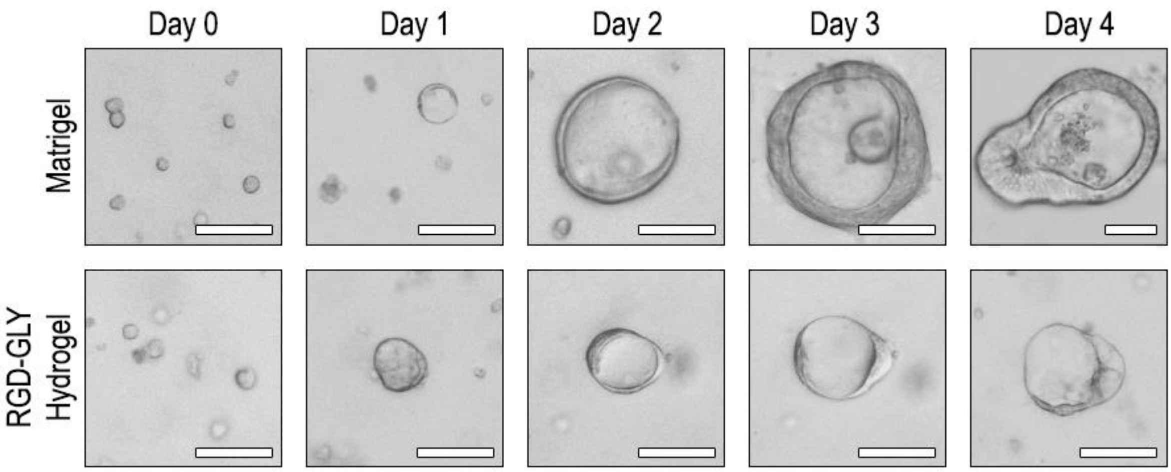

Figure S8: Organoids formed from single cells. Organoids were formed from single cells in Matrigel and RGD-GLY hydrogel and cultured during 4 days. Results shown represent independent experiments performed in triplicates (n=3). Scale bars: 100 µm.

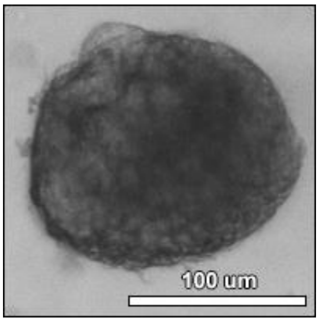

Figure S9: Small intestinal organoid in GLY hydrogel. Small intestinal crypt was cultured in GLY hydrogel, remaining in the cystic phase after 7 days. Results shown represent independent experiments performed in triplicates (n=3).

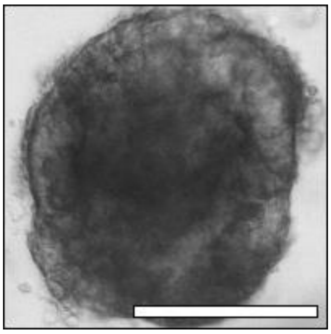

Figure S10: Cystic small intestinal organoid in RGD-GLY hydrogel. Small intestinal crypt was cultured in RGD-GLY hydrogel, remaining in the cystic phase after 4 days. Results shown represent independent experiments performed in triplicates (n=3). Scale bar = 100 µm.

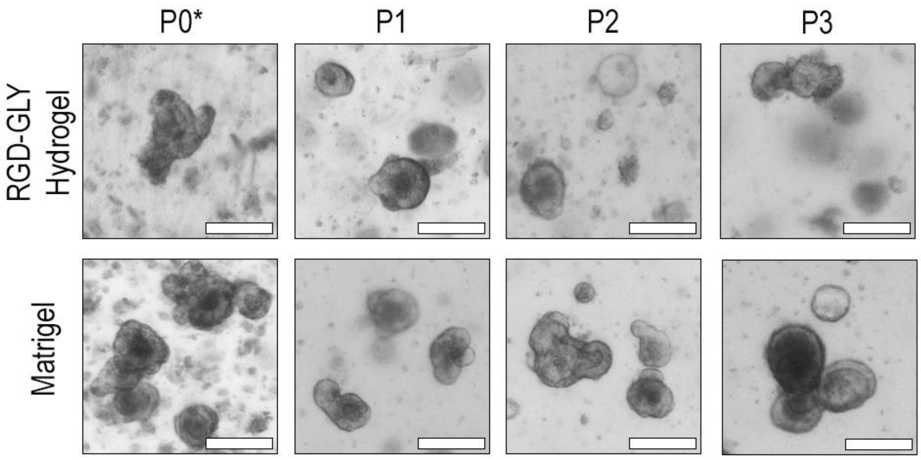

Figure S11: Small intestinal organoids passaged in RGD-GLY hydrogel and Matrigel. Organoids were passaged every 4 days during 2 weeks. Results shown represent independent experiments performed in triplicates (n=3). * = For the establishment of organoids from dissected crypts (P0), RGD-GLY hydrogel was supplement with Matrigel – 20% (v/v).

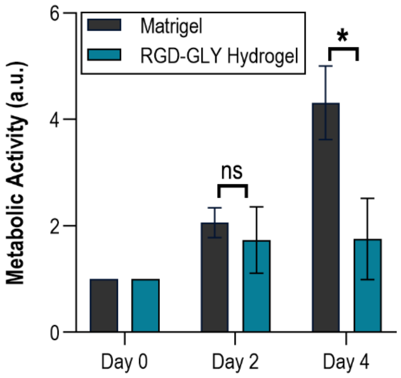

Figure S12: Measurement of metabolic activity in organoids embedded in Matrigel and RGD-GLY hydrogel. Results shown represent independent experiments performed in triplicates (n=3, error bars = SD). * = $p < 0.05$. ns = non-significant.

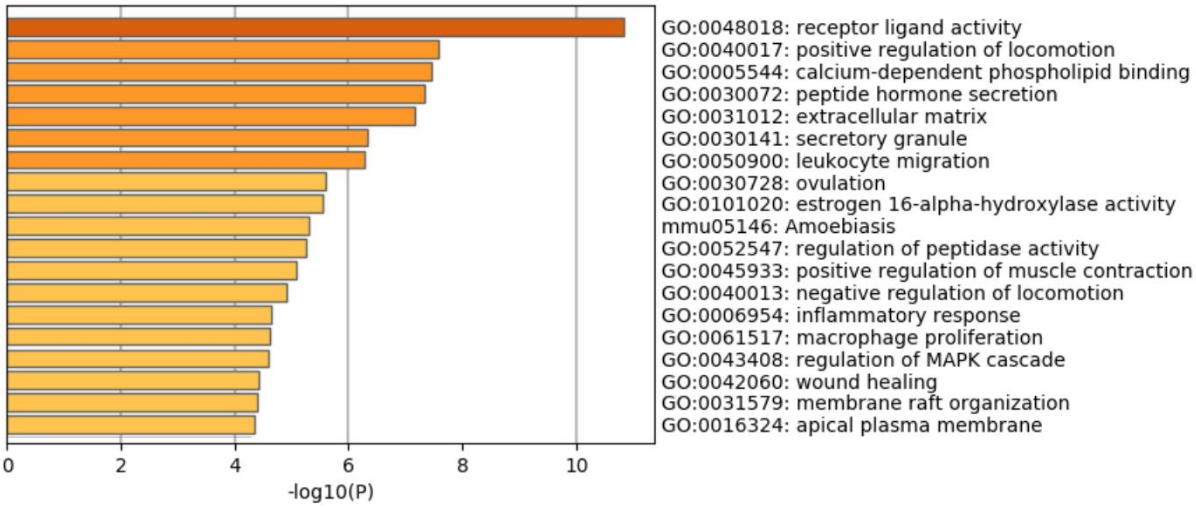

Figure S13: Gene ontology (GO) enrichment of 198 DEGs of organoids cultured in RGD-GLY hydrogel. Genes are sorted by the number associated with the listed GO ID and by descending order of –Log10 (p-value) for the GO enrichment.

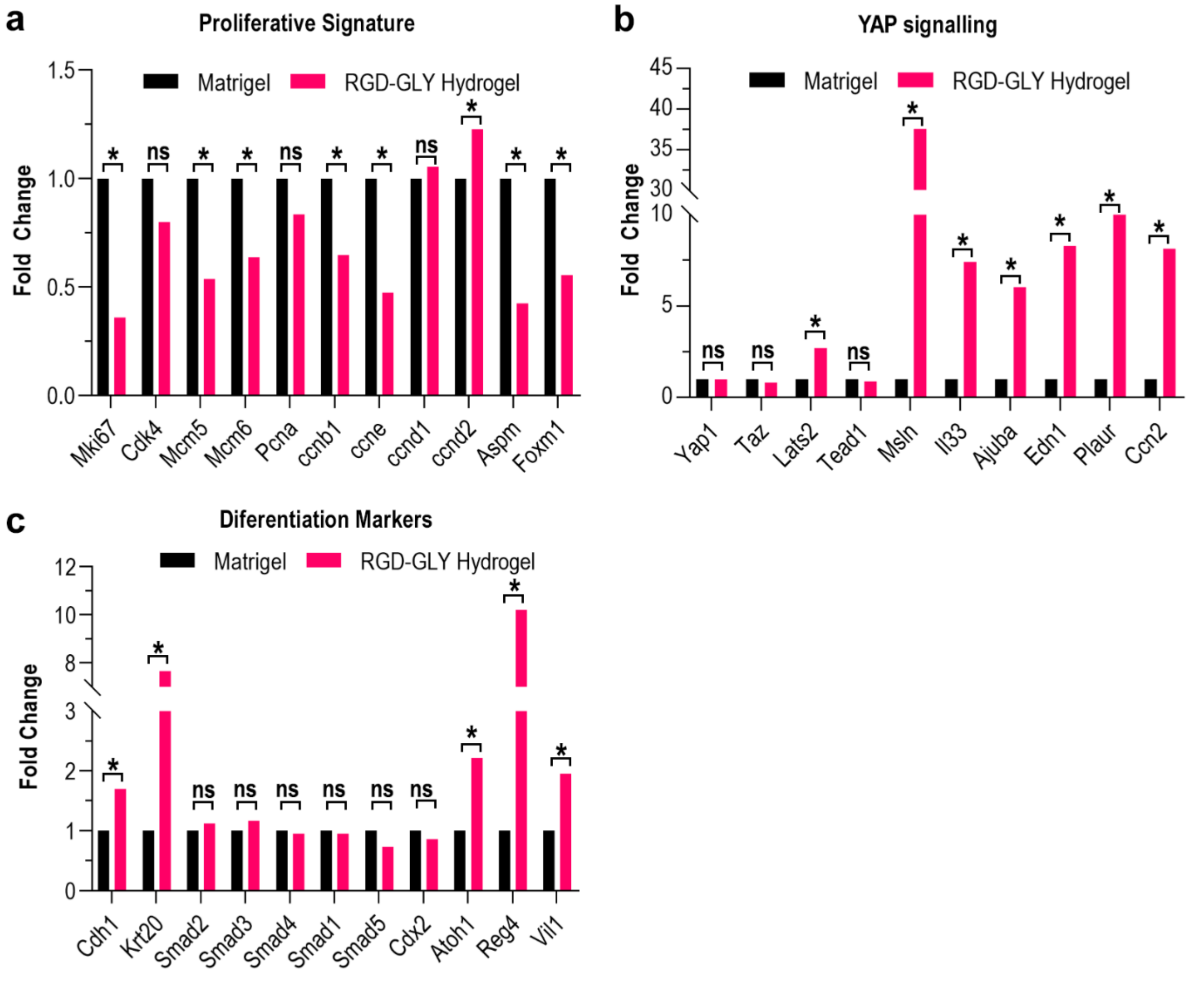

Figure S14: Gene expression in organoids cultured in Matrigel and RGD-GLY hydrogel. (A) Proliferative Signature, **(B)** YAP signaling and **(C)** Differentiation markers. Results shown represent independent experiments performed in triplicates (n=3). * = Fold change > 2, FDR and $p < 0.05$. ns = non-significant.

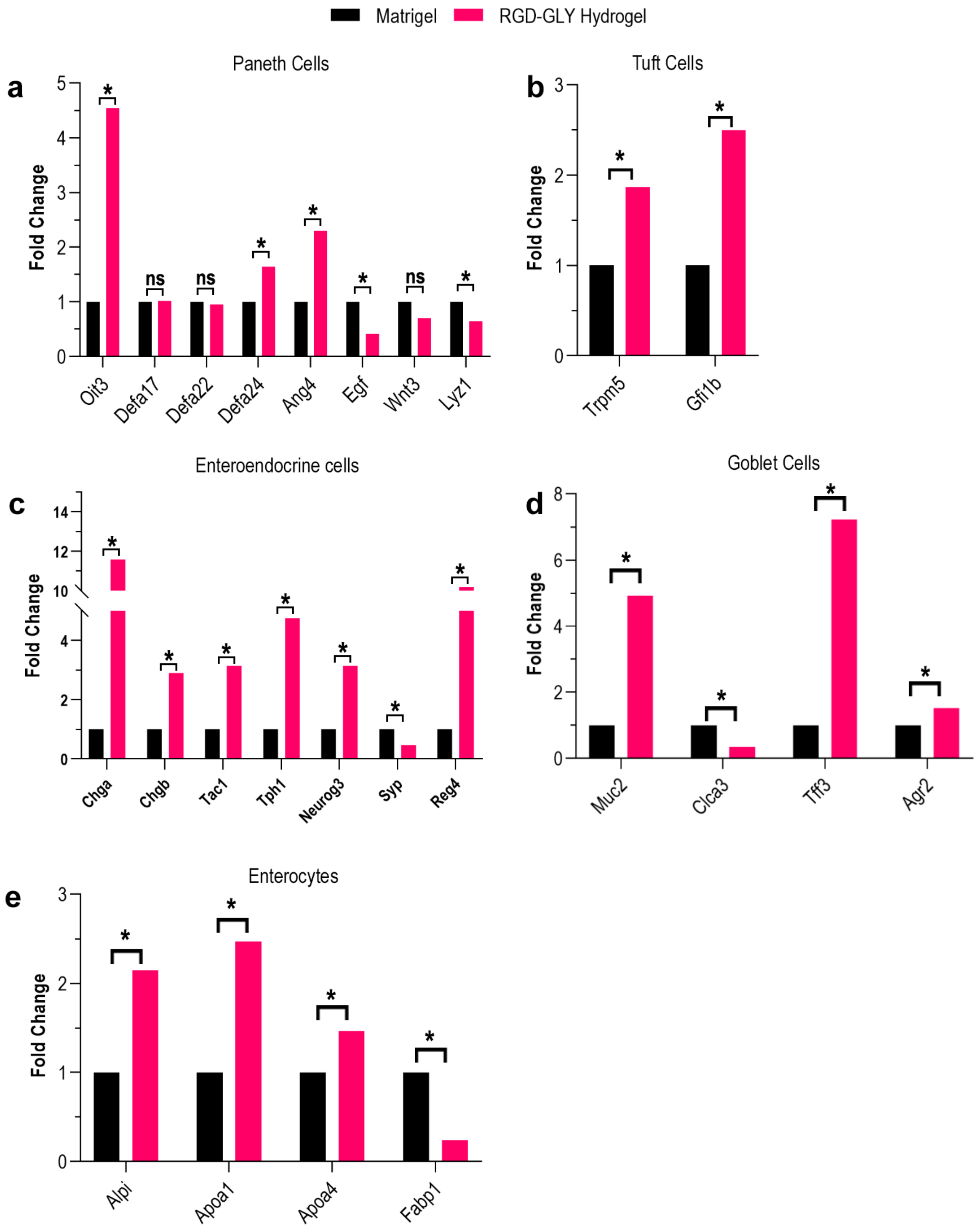

Figure S15: Gene expression in differentiated cells in organoids cultured in Matrigel and RGD-GLY hydrogel. (A) Paneth cells, **(B)** Tuft cells, **(C)** Enteroendocrine cells, **(D)** Goblet cells and **(E)** Enterocytes. Results shown represent independent experiments performed in triplicates (n=3). * = Fold change > 2, FDR and $p < 0.05$. ns = non-significant.

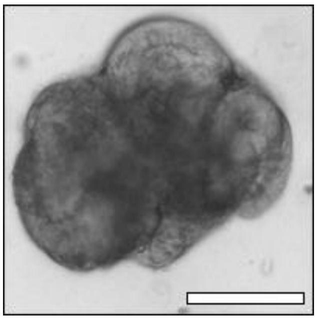

Figure S16: Small intestinal organoid in RGD-GLY matrix. Small intestinal crypt was cultured in RGD-GLY hydrogel without IGF-1 during 5 days. Scale bar: 100 µm. Results shown represent independent experiments performed in triplicates (n=3).
